# Anorexia Nervosa and the Immune System—A Narrative Review

**DOI:** 10.3390/jcm8111915

**Published:** 2019-11-08

**Authors:** Dennis Gibson, Philip S Mehler

**Affiliations:** 1 Assistant Medical Director, ACUTE Center for Eating Disorders @ Denver Health; Assistant Professor of Medicine, University of Colorado School of Medicine; 777 Bannock St., Denver, CO 80204, USA; 2 President, Eating Recovery Center; Founder and Executive Medical Director, ACUTE Center for Eating Disorders @ Denver Health; Glassman Professor of Medicine, University of Colorado School of Medicine; 7351 E Lowry Blvd, Suite 200, Denver, CO 80230, USA; Philip.mehler@dhha.org

**Keywords:** anorexia nervosa, eating disorders, immune system, inflammation, cytokines

## Abstract

The pathogenesis of an increasing number of chronic diseases is being attributed to effects of the immune system. However, its role in the development and maintenance of anorexia nervosa is seemingly under-appreciated. Yet, in examining the available research on the immune system and genetic studies in anorexia nervosa, one becomes increasingly suspicious of the immune system’s potential role in the pathophysiology of anorexia nervosa. Specifically, research is suggestive of increased levels of various pro-inflammatory cytokines as well as the spontaneous production of tumor necrosis factor in anorexia nervosa; genetic studies further support a dysregulated immune system in this disorder. Potential contributors to this dysregulated immune system are discussed including increased oxidative stress, chronic physiological/psychological stress, changes in the intestinal microbiota, and an abnormal bone marrow microenvironment, all of which are present in anorexia nervosa.

## 1. Introduction

The importance of the immune system in the pathogenesis of a large number of diseases is being increasingly accepted. Although its contribution toward organic disease is easily appreciated, the realization that the immune system is also capable of contributing to the pathogenesis of mental health disorders has only recently become more recognized as the effects of inflammation on the central nervous system function have been discovered [1]. Furthermore, research has identified significant pleiotropy between the immune system and mental health disorders [2]. However, the impact of inflammation toward the development and maintenance of anorexia nervosa has not been elucidated at this time. Anorexia nervosa, a mental illness characterized by extreme weight loss due to restricted intake resulting from an extreme fear of weight gain, ultimately impacts every organ system and has a very high recidivism rate due to the lack of efficacious treatment options. If indeed anorexia nervosa is associated with a pro-inflammatory state, as this paper will attempt to argue, weight restoration, an essential component of treatment, then becomes that much more difficult due to the hunger suppressing and weight loss effects associated with the pro-inflammatory cytokines. This paper will attempt to argue for this pro-inflammatory state by summarizing the research findings of the immune system in anorexia nervosa and how they compare to the findings in primary malnutrition. However, much of the current research on this topic is of lower quality, and this paper is not meant to serve as a systematic review. With that said, a review of the majority of the publications on the immune system in anorexia nervosa has been attempted. 

One first becomes intrigued by the possibility of a dysregulated immune system in anorexia nervosa when examining the frequency of infection in patients with anorexia nervosa. Although anorexia nervosa is a subtype of malnutrition, individuals with anorexia nervosa curiously may not suffer the same increased incidence of infection as found in other types of malnutrition [3]. A pro-inflammatory state is suggested when comparing the immunologic findings in anorexia nervosa, a state of starvation secondary to a primary mental health disease, to those present in primary malnutrition (that are related to inadequate energy intake and not secondary to another condition such as cancer, infection, malabsorption, etc.). 

Genome wide association studies, which are non-biased studies examining the entire genome for genetic variations occurring more frequently in a certain population, provide additional evidence for a dysregulated immune system that is potentially involved in the pathogenesis of anorexia nervosa. One locus found to be significantly affiliated with anorexia nervosa is associated with multiple autoimmune disorders [4]. Research has indeed found an increased association between anorexia nervosa and various autoimmune diseases, with a bidirectional relationship [5,6,7]. In addition, individuals with auto-inflammatory disease (when the innate immune system attacks various host tissues) are at a higher risk for the development of an eating disorder [6]. Curiously, there is a case report of an individual with long-standing juvenile idiopathic arthritis and anorexia nervosa who exhibited improvement in body weight and appetite after treatment with infliximab (anti-TNF therapy) [8]. There is another case report of an individual with a 12-year history of anorexia nervosa pre-dating by many years a diagnosis of Crohn’s disease, who experienced significant weight gain and no further relapse in psychopathology several months after beginning immunosuppressive therapy [9].

Another genetic finding approaching significance in anorexia nervosa involves a locus containing early B cell factor 1, which encodes a transcription factor important for immune system development, regulation of adipocyte/osteoblast differentiation and possible interaction with leptin signaling [10]. A region on chromosome 7, which includes various taste receptor genes, but also a gene important for cell–cell adhesion, apoptosis, and the immune response to pathogens, was also found to approach significance in the anorexia nervosa population [11]. 

After comparing the immunologic findings in primary malnutrition to those found in anorexia nervosa, suspicion arises as to why these differences exist. Potential contributors to these immunologic differences between primary malnutrition and anorexia nervosa will then briefly be discussed. First, a brief review of the immune system is warranted [12,13].

## 2. The Innate Immune System Overview

The innate immune system is largely composed of dendritic cells (DC), monocytes, macrophages, neutrophils, natural killer cells (NK), and the complement factors. These cells serve as the first line of nonspecific defense against potential pathogens, and are constantly surveilling the human body, recognizing highly conserved molecules on pathogens. Identification of these structural motifs by pathogen recognition receptors (i.e., Toll-like receptors) on immune system cells stimulates phagocytosis or activates other aspects of the immune system, with the ultimate goal being pathogen destruction. Complement is also capable of binding these non-host molecules, thereby marking the pathogen for destruction by phagocytes and initiating the inflammatory cascade. In addition, NK cells are capable of pathogen recognition via Toll-like receptors, and they destroy pathogens through apoptosis. However, these NK cells differ from other immune cells in that their cell killing must first be downregulated through binding to host antigens. 

Macrophages and neutrophils are both capable of phagocytosis. Activation of these cells leads to upregulation of various transcription factors that, in turn, leads to the production of various pro-inflammatory genes. Cytokines, substances that induce an inflammatory response through communication with various immune cells, aid in the recruitment of other immune cells through chemotaxis, increase vascular permeability, and perform multiple other actions on the immune system are released from the activated immune cells and largely serve to alter cell behavior. 

## 3. The Adaptive Immune System Overview

The adaptive immune system is composed of cell-mediated immunity, which involves the T cell response, and humoral immunity, which involves the B cell response. The adaptive immune system is more specific for pathogens, can take days to weeks to mount a response given use of immunologic memory, and comes into significant play when the innate immune system is incapable of controlling the reproduction of pathogens. The adaptive immune system becomes activated in lymph nodes when B cells, DCs, and macrophages present proteins derived from pathogens to cells of the adaptive immune system. 

The cell-mediated immune response is highly dependent upon T cells. T lymphocytes are produced in the marrow and mature in the thymus gland. T cells can be divided into cytotoxic T cells (CD8) and helper T cells (CD4), which can be further divided into Th1 and Th2 type cells. Naïve T cells become activated when their receptors, which are highly specific for a particular protein, bind to antigen presenting cells such as DCs and macrophages. Activated cytotoxic T cells are then capable of pathogen killing by recognizing very specific proteins located on the pathogen. CD4 cells will differentiate into Th1 cells or Th2 cells depending upon the local cytokine milieu. Naïve T helper cells favor Th1 differentiation in the presence of interleukin (IL)-12 and interferon (IFN)-γ and Th2 differentiation in the presence of IL-4. The Th1 helper cells then produce increased amounts of IL-2 and IFN-γ, favoring cell-mediated immunity, while the Th2 helper cells produce increased amounts of the cytokines IL-4, IL-5, IL-10, and IL-13, favoring humoral immunity. In general, the Th1 response is more pro-inflammatory than the Th2 response. Naïve helper T cells can also differentiate into T regulatory cells, which serve to downregulate the immune response. 

Cell-mediated immunity is largely tested through delayed cutaneous hypersensitivity (macrophage interaction with CD4 helper T cells causes the release of Th1 cytokines, which then recruit and activate cytotoxic T cells, and these cells attempt to destroy the antigen with a localized immune response that can be observed at the skin) and lymphocyte proliferation (a measure of clonal expansion of T cells after exposure to antigen). 

The humoral immune response is dependent upon the production of antibodies by B cells. Naïve helper T cells differentiate into Th2 cells upon binding antigen presenting cells and in the presence of certain cytokines. These Th2 cells then bind B cells specific for certain antigens, thereby activating the B cells and increasing immunoglobulin production specific for that pathogen. Once the immunoglobulins are secreted, they are capable of binding directly to the pathogen or to complement, increasing phagocytosis and stimulating further inflammation. 

## 4. The Immune System in Primary Malnutrition

The lymphatic tissues in animals and children suffering from primary malnutrition exhibit significant histologic changes. The thymus shows generalized atrophy and distorted architecture [14,15,16]. The peripheral lymph nodes and spleen also show generalized atrophy and distorted architecture [14,15,16], although to a lesser extent than that seen in the thymus [14,15]. The bone marrow frequently becomes hypo-cellular with decreased hematopoietic stem cells (HSC) (the precursor to the hematologic cells produced in the marrow) and increased adipocytes [17,18,19], contributing to lymphopenia, decreased myeloid cells (neutrophils and monocytes), and potentially other plasma hematologic changes (i.e., anemia) as found in murine models [17,20,21]. However, plasma leukocyte and lymphocyte levels in children with malnutrition are conflicting, likely related to recent or concomitant infection as well as the methodology employed [22,23]. Nonetheless, research suggests that leukopenia is present in human subjects when malnutrition is not associated with other processes such as infection [24,25].

Significant deficits in the functioning of the innate immune system are suggested in primary malnutrition. Neutrophil chemotaxis (cellular movement toward a stimulus) seems to be abnormal [15,23,26]. Neutrophil intracellular killing with various enzymatic activities is also likely to be impaired [15,23,27]. Results of phagocytosis are, however, contradictory [15,23] and may be related to whether activated neutrophils obtained from sites of inflammation versus neutrophils circulating in plasma are being studied [26,28]. Macrophage function including chemotaxis, phagocytosis, and intracellular killing all seem to be largely impaired in primary malnutrition [15,20,29]. Complement levels and function appear to be decreased [15,23,30,31], and DC function may be impaired [32]. In addition, NK cell cytotoxicity seems impaired in a minority of individuals with malnutrition, although collectively it is not statistically decreased [33,34]; it is likely that those select individuals with impairment are suffering from various micronutrient deficiencies [35,36,37]. 

Studies in primary malnutrition are also suggestive of abnormalities with cell-mediated immunity [38], resulting in a reduced cytotoxic T cell response [39] and reduced delayed cutaneous hypersensitivity [16]. The ratio of helper to cytotoxic T cells (CD4/CD8) seems abnormally low in this population [40,41]. CD4 counts seem to be more greatly decreased than CD8 counts in malnutrition when infection is not present [39,42]. Results examining lymphocyte proliferation depend on the antigen used, although this appears to be overall impaired in primary malnutrition [22,23]. 

Studies examining cytokine production in primary malnutrition can be contradictory based on the methodology used (in vitro or in vivo), but certain patterns are nonetheless suggested. Cytokine production in malnutrition favors the Th2 pathway with overall increased production of IL-4 and decreased production of IL-12 and IFN-gamma [23,43,44,45,46]. Stimulated and spontaneous production of IL-1 suggest either unchanged or decreased concentration [20,47,48,49,50]. Interpretation of IL-6 production in malnutrition is made difficult by the findings of decreased, unchanged, and elevated levels when compared to the controls; however, when controlling for infection, IL-6 seems to be overall decreased [20,45,47,48,51,52,53]. Studies also seem to suggest decreased stimulated production of tumor necrosis factor-alpha (TNF) when controlling for infection [20,49,51,53,54], without any suggestion of increased spontaneous release of TNF [49,55].

Abnormalities in humoral immunity are quite controversial in the setting of primary malnutrition due to limited research, but certain patterns are suggested. Diminished lymphopoiesis leads to decreased circulating B cells [15,23,56]. However, B cell function appears mostly intact when controlling for infection [15,22,23]. Studies are suggestive of decreased secretory IgA levels [15,23,57], which are important for mucosal immunity, but otherwise preserved immunoglobulin production and concentration (although it remains possible that these antibodies may have lower affinity to antigens) [58,59]. Research also suggests that abnormalities in the T cell-B cell interaction contribute to abnormalities with antibody production, although this appears to be secondary to helper T cell abnormalities with intact B cell function [41,60]. 

## 5. The Immune System in Anorexia Nervosa

The lymphoreticular system (spleen, lymph nodes, thymus, and bone marrow) is largely unstudied in anorexia nervosa except for findings regarding the bone marrow, wherein there is the frequently noted condition referred to as gelatinous marrow transformation (GMT) [61,62,63]. GMT is associated with an overall hypocellular marrow with decreased adiposity, and instead, the deposition of thick amorphous gelatinous substances in the extracellular spaces [62,64], contributing to the decreased red and white cell counts seen in these patients [61]. This differs from primary malnutrition, which is frequently associated with a hypocellular marrow, but increased marrow adiposity [17].

The innate immune system in anorexia nervosa has multiple abnormalities. A single study of ten individuals suffering from anorexia nervosa found deficits in neutrophil chemotaxis when compared to a control group of expanded size (*p* < 0.05, *n* = 44), with chemotaxis nearly absent in two patients with anorexia nervosa; neutrophil adherence was also decreased when compared to the controls (*p* < 0.001) [65]. Defects in granulocyte microbicidal activity were also suggested in one study, who found decreased alkaline phosphatase in five of six patients with anorexia nervosa [65]. Although limited in sample size (*n* = 3), another study found reduced ability of granulocytes to kill two bacterial species [66]. Similarly, neutrophil phagocytosis is poorly studied in anorexia nervosa, consisting of only a single small study (*n* = 3) that found intact opsonization with Staphylococcus aureus [66]; however, no studies have been completed on the phagocytic function of activated neutrophils obtained from sites of inflammation. Serum complement C3 levels were found to be decreased in anorexia nervosa compared to the controls (*p* < 0.001) in a small study (*n* = 14), but 50% hemolytic complement activity (CH50) was not statistically different [67]. Similarly, serum complement C3 (*p* < 0.001), along with C1q (*p* < 0.05) and C2 (*p* < 0.001), were all low in the anorexia nervosa group of another study (*n* = 14), but with normal serum levels of C4, C5, and C6 when compared to the controls [67]. Furthermore, C3 levels appear to correlate with nutritional status, improving with weight restoration [67,68]. NK cell quantity is reduced in anorexia nervosa when compared to the controls [69,70,71], but NK cell activity seems intact based on the few studies completed [72,73]. DC and macrophage function in anorexia nervosa are unstudied. These aforementioned findings of the innate immune system in patients with anorexia nervosa are thus similar to those noted in primary malnutrition.

Cell-mediated immunity in anorexia nervosa appears to be “dysregulated” when compared to the immunologic abnormalities observed in primary malnutrition. Nine patients with anorexia nervosa had insignificant skin reactions to various mitogens; however, four individuals were unresponsive (anergic) to the mitogen [74]. In addition, greater mitogen concentrations were required to elicit a similar reaction to the controls (*p* < 0.005), although still dependent on the mitogen used [74]. A study of 22 individuals with anorexia nervosa found anergy in six individuals, with five of these individuals weighing less than 60% of their ideal body weight [75]. Similarly, a study of 12 individuals with anorexia nervosa examining cell-mediated cytotoxicity found a significantly reduced response when compared to the controls (*p* < 0.05) [76]. T cell proliferation appears overall intact, if not increased, though still dependent upon the mitogen used [77,78,79,80]. Nagata et al. [77] and Silber et al. [78] reported similar responses to various mitogens when comparing individuals with anorexia nervosa to a control group. However, Golla et al. [79] and Bentdal et al. [80] both reported statistically significant increased T cell responsiveness, although dependent upon the mitogen used. Overall, these results suggest diminished delayed type hypersensitivity and cell-mediated cytotoxicity, similar to primary malnutrition. However, T cell proliferation seems intact, if not exaggerated, compared to the response observed in primary malnutrition. 

T cell subtypes also appear to be “dysregulated” when comparing anorexia nervosa to primary malnutrition. The CD4/CD8 ratio in anorexia nervosa is seemingly increased, and this appears due to a greater reduction in CD8 counts compared to CD4 counts [70,77,81,82]. Elegido et al. [70] and Mustafa et al. [81] both attributed this abnormality in CD8 counts to a statistically significant decrease in memory CD8 cells as opposed to naïve CD8 cells (*p* < 0.01). Nagata et al. [77] also found greater elevation in the CD4/CD8 ratio with more significant weight loss (*p* < 0.05); indeed, these researchers suggest that with greater depletion in body weight, lymphocyte production is prioritized over other immune cells, especially naïve T helper cells, in the attempt to preserve the efficacy of the adaptive immune system [77]. Regulatory T cell function seems to be unaffected in anorexia nervosa based on a single study [83]. 

Fewer studies have been completed with regard to the humoral system in anorexia nervosa and are inconclusive. One study with 16 individuals suffering from anorexia nervosa found normal serum IgA, IgM, and IgG [84]. However, another small study (*n* = 5) found decreased IgG and IgM when comparing anorexia nervosa patients to healthy controls [67]. One study found only reduced IgG when comparing individuals with greater severity of anorexia nervosa (BMI less than 17.5) to the controls (*p* < 0.05) [69]. One study seems to suggest normal B cell counts [69], while another study found increased B cell counts in 46 anorexia nervosa patients when solely examining the restrictive subtype; indeed this population suffered greater weight loss than the individuals in the binge/purge subtype, and negative correlation between B cell counts and BMI was also found in this study [70]. T–B cell interaction has also been found to be abnormal in this population based on a single study [69]. 

Therefore, abnormalities in the innate immune system are largely similar between anorexia nervosa and primary malnutrition. Although a few differences seem to exist regarding cell-mediated immunity including potentially enhanced T cell proliferation in anorexia nervosa compared to primary malnutrition, and increased CD4/CD8 ratios in anorexia nervosa compared to primary malnutrition, the findings are largely similar between anorexia nervosa and primary malnutrition. Conclusions regarding the similarities and differences between the humoral immune system in anorexia nervosa and primary malnutrition are more difficult to determine with the current research.

These above aberrations in the immune system in anorexia nervosa and primary malnutrition are likely multifactorial, albeit expected, given the significant interaction between the state of nutrition and immune system function [85,86]. Leptin is an adipokine (secreted by adipocytes), similar in structure to multiple cytokines, that circulates at levels correlating with the density of adipose tissue and nutritional status. Adequate leptin levels are indeed needed for nearly all aspects of the immune system to function properly including cellular proliferation of the thymus and peripheral lymphoreticular system [14]; bone marrow cellular proliferation and hematopoiesis [87,88,89]; macrophage phagocytosis, chemotaxis, and microbicidal activity [86,90,91,92]; neutrophil chemotaxis and microbicidal activity [86,90]; complement function [93]; increasing CD4 T cell proliferation [94,95]; Th1 cytokine response (inadequate leptin favors Th2 response) [86,90,96,97]; cytotoxic T cell activity [90]; survival of B cells [56,86,90]; and NK cell proliferation [86]. The immune system changes in primary malnutrition can likely be explained by a leptin deficiency, which is low in primary malnutrition. Furthermore, serum leptin levels are known to be abnormally low in anorexia nervosa [98]. Moreover, with weight restoration in patients with anorexia nervosa, leptin levels rise back to a normal range. One must, therefore, question the etiology of the possibly increased CD4/CD8 ratios and intact lymphoproliferative response noted in anorexia nervosa when compared to primary malnutrition. 

Furthermore, an examination of the cytokine profile in anorexia nervosa suggests a “dysregulated” immune system when compared to the cytokine profile present in primary malnutrition. Although the Th2 pathway seems to be favored in anorexia nervosa and primary malnutrition, with increased IL-4 as well as decreased IL-2 production [69,99,100], pro-inflammatory cytokines appear to be upregulated in anorexia nervosa. Studies examining IL-6 [82,100,101,102,103], IL-1 [100,101,104,105], and IFN-γ [101,102,106,107] have found decreased, unchanged, or increased levels, depending on the methodology employed. However, one meta-analysis was suggestive of overall increased IL-1 and IL-6 in anorexia nervosa when compared to healthy controls (*p* = 0.003 and *p* = 0.009, respectively) [106], while another meta-analysis reported increased IL-6 when compared to the controls (*p* = 0.001) [107], but elevated IL-1 only when comparing the restricting subtype of anorexia nervosa to the controls (*p* = 0.018); there was no statistical significance in this meta-analysis when comparing all subtypes of anorexia nervosa to healthy controls (*p* = 0.110).

Studies examining tumor necrosis factor (TNF) levels have also resulted in decreased, unchanged, or increased levels of this cytokine [55,69,76,100,101,102,103,104,108,109], but a majority of the studies suggest increased secretion of TNF by immune cells in anorexia nervosa [55,69,76,100,101,103,104,109]. Indeed, meta-analyses have found elevated levels of TNF in anorexia nervosa when compared to the controls (*p* = 0.008, *p* = 0.015) [106,107]. Furthermore, research is suggestive of increased spontaneous production of TNF from circulating monocytes and lymphocytes in individuals with anorexia nervosa when studied in vitro [55,69,76]. One study, directly comparing spontaneous TNF production in seven patients with anorexia nervosa and six patients with primary malnutrition (infection free), found significantly greater levels in anorexia nervosa when compared to primary malnutrition (*p* < 0.0006) [55]. In addition, the current research suggests that mRNA levels of TNF might remain elevated with refeeding and weight restoration, although other cytokines seem to normalize [104,106]. 

To summarize, there are similarities between anorexia nervosa and primary malnutrition, but there also exist the following immune system differences between anorexia nervosa and primary malnutrition: (1) anorexia nervosa is associated with the bone marrow changes of GMT and reduced marrow fat, which are not also seen in primary malnutrition; (2) T cell proliferation to various antigens appears decreased in primary malnutrition when compared to the response in anorexia nervosa; (3) CD8 cell counts seem to be more affected in anorexia nervosa without similar affects noted in primary malnutrition; (4) various pro-inflammatory cytokines (IL-1, IL-6, and TNF) seem to be elevated in anorexia nervosa when compared to levels in primary malnutrition (when adequately controlled for infection); and (5) there may be increased spontaneous production of TNF in anorexia nervosa that is not present in primary malnutrition (see Table 1). 

Therefore, one is left to ponder the question as to the cause of these immunologic differences between anorexia nervosa and primary malnutrition if working under the assumption that the immune system changes are solely due to malnutrition. Furthermore, these changes may suggest a pro-inflammatory state in anorexia nervosa that does not appear to be present in primary malnutrition. The genetic studies discussed above suggest there may be some intrinsic abnormality within the immune system that contributes to this pro-inflammatory state; however, this remains speculative. Several potential contributors to this “dysregulated” immune system in anorexia nervosa are discussed below, although their contributions are highly speculative at this time. These include increased oxidative stress, a chronically activated sympathetic nervous system (SNS) and hypothalamic-pituitary-adrenal (HPA) axis, altered intestinal microbiota, and an abnormal bone marrow microenvironment. 

## 6. Oxidative Stress 

One potential contributor to an upregulated immune system in anorexia nervosa is increased free radical formation causing increased oxidative stress [110]. Briefly, free radicals are atoms with unpaired electrons, making them highly unstable and capable of causing damage to all tissues by disrupting cellular function. In the human body, reactive oxygen species (ROS) are constantly generated in mitochondria as electrons flow down the electron transport chain through the process of aerobic metabolism. Important sources of increased oxidation in anorexia nervosa include not only the aerobic metabolism of nutrients, but also the activation of the arachidonic acid cascade and activation of various enzymes used by phagocytes in pathogen killing [111]. Once generated, these ROS are capable of activating the inflammatory cascade and increasing production of pro-inflammatory cytokines [110]. This is largely accomplished through lipid peroxidation, which occurs when free radicals interact with cell membrane fatty acids, disrupting the lipid structure and thereby altering downstream intracellular signaling as well as propagating additional damage to proteins, DNA, and other cellular structures [112]. 

Individuals with anorexia nervosa have increased oxidative stress [113,114,115,116], partially attributed to decreased levels of anti-oxidants [113,117,118]. Studies in this population have also found abnormalities in the electron transport chain, likely further contributing to increased oxidative stress [119,120]. Changes in lipid peroxidation also contribute [121], as individuals with anorexia nervosa have various fatty acid deficiencies including the omega 3 fatty acids that have anti-inflammatory properties [122,123,124]. By increasing the ratio of omega 6 to omega 3 fatty acids within the cell membrane (the typical Western diet contains much greater amounts of omega 6 than omega 3 fatty acids), increased amounts of arachidonic acid are produced, thereby increasing the pro-inflammatory secondary signals [125]. 

Refeeding, critical to the sustained recovery of patients with anorexia nervosa, is another potential contributor to the upregulated immune system presumed in this condition. Although unstudied in eating disorders, oxidative stress is increased in animal models of starvation when undergoing refeeding and may potentially be related to increased adiposity (and associated adipokine secretion) with weight gain [126]. Increased central adiposity has indeed been found in anorexia nervosa with weight restoration [127], and this regional adiposity is very metabolically active [128]. Furthermore, “postprandial dysmetabolism”, which is a function of increased oxidative stress following food consumption due to elevated plasma glucose and lipids, creates increased inflammation for several hours following oral consumption [129,130]. This dysmetabolism is believed to be secondary to the effects of glucagon [129]. Although postprandial dysmetabolism has not been directly studied in anorexia nervosa, individuals with anorexia nervosa behave similarly to diabetics before weight restoration in that they have higher plasma glucagon and greater plasma glucose levels during a glucose tolerance test [131], supporting a role for “postprandial dysmetabolism” in this population.

## 7. Chronic Stress 

Stress, “a set of constructs representing stages in a process by which environmental demands that tax or exceed the adaptive capacity of an organism occasion psychological, behavioral, and biological responses that may place persons at risk for disease” [132], is another potential contributor to the upregulated immune system in anorexia nervosa. Both physiologic stressors and situations perceived as stressful are capable of activating the hypothalamic-pituitary-adrenal (HPA) system and sympathetic nervous system (SNS), ultimately resulting in increased cortisol and norepinephrine (NE)/epinephrine (EPI), respectively, and thereby affecting the immune system in anorexia nervosa. Cortisol affects the immune system through binding with glucocorticoid receptors, which downregulate gene transcription of inflammatory mediators. NE and EPI act through the mostly anti-inflammatory β2 receptors present on the innate and adaptive immune cells, although α1 receptors are also present and tend to upregulate the immune system. Under homeostatic conditions, the β2 receptor mediated signals predominate [133]. 

Research supports both pro- and anti-inflammatory effects with long-term stressors [134,135]. However, it is being increasingly suggested that the target tissue response is more important than circulating hormone levels [135]. Indeed, studies have found glucocorticoid receptor desensitization with long-term activation of the HPA system, mitigating the anti-inflammatory response to cortisol [134,135,136]. Although not as well studied and the implications of these findings are unclear, a similar mechanism is suggested for the long-term activation of the SNS. The β receptors become desensitized to NE and EPI, requiring higher levels of hormones to have the same effect [137,138]. Chronic binding of the hormone to the β receptors also causes them to become internalized [137], thereby increasing the concentration of the pro-inflammatory α1 receptors on the cell membrane. In addition, chronic activation of the β receptor alters intracellular signaling toward a pro-inflammatory state [137,139]. 

When examining target tissue response in those with anorexia nervosa, the findings are suggestive of increased activation of the stress response systems. Individuals with anorexia nervosa lose responsiveness to glucocorticoid stimulation [140,141,142], and decreased numbers of β adrenergic receptors have been found on immune cells [143], both of which would contribute to a pro-inflammatory state. 

Furthermore, individuals with anorexia nervosa have high co-morbidity of obsessive compulsive disease, anxiety, depression, post-traumatic stress disorder, and other mental health disorders that are associated with a chronically upregulated HPA axis and SNS [144]. Depression has been found to be associated with abnormal β adrenergic responsiveness [145], anxiety is associated with downregulation of the β adrenergic receptors [146], and panic disorder is associated with decreased β adrenergic receptors on cell membranes as well as a reduced response to β agonists [147]. 

Although it would seem that chronic stress impacts immune function, the above discussion is overly simplified. Thus, the true effects of stress on immune function in anorexia nervosa and the comorbid illnesses are unknown. The other hormones/cytokines present in the local milieu of the immune cells, the timing of the stressor(s) on immune cell development, the type of immune cells studied, and whether the immune cells are obtained from lymph nodes or other sites all likely impact whether a pro- or anti-inflammatory effect is observed. 

Stress has also been found to alter intestinal permeability through the actions of corticotropin releasing hormone and mast cell activation, causing increased gut permeability and translocation of commensal and pathogenic bacteria [148,149,150]. The implications of this are discussed below. 

## 8. Intestinal Microbiota

The trillions of commensal bacteria normally inhabiting the human intestinal tract are very important for normal gut health and function and are referred to as the microbiota [151]. They play a role through the production of various metabolites such as the short chain fatty acid butyrate, which serve as nutrition for the epithelial cells of the gastrointestinal tract [152]. The commensal bacteria normally inhabiting the gastrointestinal tract are also very important for appropriate development of the immune system [153], and for maintenance of immune tolerance in this state of symbiosis [151]. However, non-commensal gut microbes have developed means to evade the mucosal barrier and interact with the intestinal epithelial cells [154]. These pathogenic bacteria are then capable of activating the immune system through multiple processes: direct activation of the immune system via the constant sampling of the luminal contents by the host’s immune cells [153], activation of the various immune receptors located directly on the gut epithelial cells [153], loss of commensal bacterial metabolites that downregulate the immune response through cell signaling [155], and disruption of the intestinal barrier [156], leading to a proclivity toward sepsis and gastrointestinal infections. Increased production of pro-inflammatory cytokines by the epithelial and immune cells further disrupts the intestinal barrier [157], and a recurring pattern then develops [158]. 

The role of the microbiota is being increasingly studied in anorexia nervosa; however, it is beyond the scope of this article to adequately discuss the microbiota and how it relates to anorexia nervosa. For a good review on the topic of the microbiota and anorexia nervosa, see Roubalova et al. [159]. Briefly, microbiota changes do seem to occur in anorexia nervosa with weight loss and weight restoration [160,161,162,163] as well as decreases in short chain fatty acid production [161]. Weight loss alone is also capable of disrupting the intestinal epithelial barrier [158]. All of these changes would be expected to ultimately lead to upregulation of the immune system. However, one study examining intestinal permeability in anorexia nervosa actually found decreased intestinal permeability [164]. Ultimately, it is currently unknown how the microbiota changes impact the immune system in anorexia nervosa and is only speculative that these changes impact the functioning of the immune system. 

## 9. Gelatinous Marrow Transformation and Mesenchymal Stem Cells

An appropriately functioning bone marrow is necessary not only for adequate hematopoiesis, but also for regulation of immune cell activity [165]. The mesenchymal stem cells (MSC), which are the precursors for the marrow stromal compartment, regulate growth and differentiation of hematopoietic stem cells, and have an immunomodulatory effect on the various immune cells [166,167]. Local cytokines [165,168,169], SNS input [170,171], and MSC interactions with other cells in the marrow [165,170] all impact factors produced by the MSCs. For example, IFN-γ appears to be the most important cytokine for “licensing” the largely immunosuppressive functions of the MSCs [166,167,172]. These MSCs are then capable of inhibiting nearly all cells of the immune system [173,174,175,176,177,178,179]. 

Although MSCs are largely immunosuppressive, they can also have immune stimulatory properties [166]. These cells are capable of phagocytosis and can have antigen presenting properties [180]. MSCs tend to lose their immunosuppressive properties with changes in the microenvironment including changes in MSC concentration [179,181], changes in various cytokine concentrations [180,182], and as a consequence of alterations in MSC interactions with other immune cells in the marrow [175]. 

The marrow microenvironment is also largely responsible for MSC differentiation into adipocytes and osteoblasts, amongst other cells [87,183]. MSC differentiation into osteoblasts is favored with exposure to various growth factors including leptin [87,184], while differentiation into adipocytes is favored with upregulation of the transcription factor peroxisome proliferator-activated receptor-γ (PPARγ) [183,184]. PPARγ appears to be upregulated in malnutrition and fasting, contributing to the increased marrow adiposity seen with malnutrition [17,185]. Similar to adipocytes throughout the rest of the body, these marrow adipocytes are capable of producing pro-inflammatory adipokines [186], which could alter the marrow microenvironment and immunomodulatory properties of the MSCs. 

Although increased marrow adiposity can be seen in anorexia nervosa, frequently, the rule is that these patients develop GMT, associated with decreased adiposity. The pathogenesis of GMT is incompletely understood, but the microenvironment is highly altered, likely due to fat mobilization and secondary hyaluronic acid deposition [187]. Although in anorexia nervosa the development of this condition is dependent upon amount of weight loss [61], weight loss is not a pre-requisite as it has also been observed in individuals with other conditions without documented weight loss such as Hashimoto’s thyroiditis, chronic heart failure, and acute severe infection [62]. Furthermore, GMT seems to be uncommon in individuals with kwashiorkor or marasmus [64], and seems to be very rare in children [159,187], even though there is a higher prevalence of primary malnutrition in this age group. Current evidence does seem to suggest that an upregulated immune response is a likely contributor toward the development of this condition [62,64,187]. Therefore, one is left to question how these bone marrow changes including a decreased amount of the bone marrow adipose tissue that is important for immune system regulation, impacts the immune system in anorexia nervosa, and, although speculative, whether this is a significant contributor to the immune system changes present in anorexia nervosa compared to primary malnutrition, given that GMT and low marrow adiposity is not noted in primary malnutrition.

## 10. Conclusions

The aforementioned increased secretion and concentration of the inflammatory cytokines as well as genetic studies strongly suggest a “dysregulated” immune system in anorexia nervosa. When comparing the immune system changes in protein malnutrition and anorexia nervosa, it is suggested that anorexia nervosa is associated with increased pro-inflammatory cytokines, an elevated CD4/CD8 ratio, and increased T cell proliferation. It is difficult to explain these immunologic changes as occurring solely secondary to malnutrition. Although the exact pathogenesis of these immunologic changes in anorexia nervosa is unclear, a potential primary immunologic defect contributing to the development of anorexia nervosa, which is possibly compounded by the conditions briefly discussed in this article, remains a strong possibility. However, this remains speculative at this time, and the contribution from oxidative stress, chronic psychological stress, an altered microbiota, and an abnormal bone marrow microenvironment, is currently unknown. Additional research must be completed to determine the etiology of the pro-inflammatory cytokine production as well as the effects of this pro-inflammatory state toward the development and maintenance of anorexia nervosa. Although heretofore anorexia nervosa was considered to have a bland state devoid of inflammation, as suggested by basic markers of inflammation such as sedimentation rates, it is becoming increasingly intriguing to consider that the immune system may actually be causal in the pathogenesis and maintenance of anorexia nervosa. 

## Figures and Tables

**Table 1:** Immune system and cytokine concentration differences between anorexia nervosa and primary malnutrition.

	Bone Marrow	T Cell Proliferation	CD4/CD8 Ratio	IL-1	IL-6	TNF
**Anorexia nervosa**	Gelatinous Marrow Transformation (GMT) (low adiposity)	Unchanged to increased	High (greater effect on CD8 cells)	Normal to increased	Increased	High (including spontaneous production)
**Primary malnutrition**	Increased adiposity without GMT	Decreased	Low (greater effect on CD4 cells)	Low to normal	Decreased	Low (no spontaneous production)
